# Development, Design and Utilization of a CDSS for Refeeding Syndrome in Real Life Inpatient Care—A Feasibility Study

**DOI:** 10.3390/nu15173712

**Published:** 2023-08-24

**Authors:** Lara Heuft, Jenny Voigt, Lars Selig, Maria Schmidt, Felix Eckelt, Daniel Steinbach, Martin Federbusch, Michael Stumvoll, Haiko Schlögl, Berend Isermann, Thorsten Kaiser

**Affiliations:** 1 Institute of Human Genetics, University Medical Center Leipzig, 04103 Leipzig, Germany; 2 Institute for Laboratory Medicine, Clinical Chemistry and Molecular Diagnostics, University Medical Center Leipzig, 04103 Leipzig, Germany; 3 Department of Endocrinology, Nephrology and Rheumatology, University Medical Center Leipzig, 04103 Leipzig, Germany; 4 Helmholtz Institute for Metabolic, Obesity and Vascular Research (HI-MAG) of the Helmholtz Zentrum München at University Medical Center Leipzig, 04103 Leipzig, Germany; 5 Institute for Laboratory Medicine, Microbiology and Pathobiochemistry, Medical School and University Medical Center OWL, Hospital Lippe, Bielefeld University, 32756 Bielefeld, Germany

**Keywords:** refeeding syndrome, CDSS, clinical nutrition, computerized decision support system, malnutrition, diagnostic support

## Abstract

Background: The refeeding syndrome (RFS) is an oftentimes-unrecognized complication of reintroducing nutrition in malnourished patients that can lead to fatal cardiovascular failure. We hypothesized that a clinical decision support system (CDSS) can improve RFS recognition and management. Methods: We developed an algorithm from current diagnostic criteria for RFS detection, tested the algorithm on a retrospective dataset and combined the final algorithm with therapy and referral recommendations in a knowledge-based CDSS. The CDSS integration into clinical practice was prospectively investigated for six months. Results: The utilization of the RFS-CDSS lead to RFS diagnosis in 13 out of 21 detected cases (62%). It improved patient-related care and documentation, e.g., RFS-specific coding (E87.7), increased from once coded in 30 month in the retrospective cohort to four times in six months in the prospective cohort and doubled the rate of nutrition referrals in true positive patients (retrospective referrals in true positive patients 33% vs. prospective referrals in true positive patients 71%). Conclusion: CDSS-facilitated RFS diagnosis is possible and improves RFS recognition. This effect and its impact on patient-related outcomes needs to be further investigated in a large randomized-controlled trial.

## 1. Introduction

The refeeding syndrome (RFS) is a complication that can occur during the reintroduction of calories in patients suffering from malnutrition or with prolonged periods of reduced food intake [1,2]. Diseases associated with malnutrition increase the risk of RFS occurrence. These include malassimilation and malabsorption syndromes, such as inflammatory bowel disease or radiation enteritis, consumptive diseases, such as cancer and HIV, psychiatric diseases, such as eating disorders and depression, as well as, for example, patients undergoing bariatric surgery or patients with severe nausea and vomiting [3]. Enteral refed patients are more likely to develop RFS [4], but any route of nutrition (oral, enteral or parenteral), as well as simple normal meals, can cause RFS. The occurrence of RFS causes broad and non-specific symptoms, such as electrolyte imbalances (specifically regarding phosphate, potassium and magnesium), fluid shifts, central and peripheral edema, cardiac arrhythmias or impaired cognitive function. A drop of serum phosphate concentration below its lower reference range (<0.84 mmol/L) during the first five days of refeeding is considered to be a hallmark RFS symptom. RFS can be fatal in patients with severe disease [5]. The underlying pathophysiology is not yet fully understood. An imbalance in the adjustment from catabolic to anabolic metabolism is thought to be the most likely causative factor of RFS [6]. During prolonged fasting, intracellular electrolyte concentrations such as potassium, phosphate and magnesium become depleted. This change is not mirrored in serum due to reduced renal excretion for hemostasis maintenance, masking the increasing shortage [7]. Physicians frequently do not recognize malnutrition and RFS in inpatients, which leads to general undertreatment and underreporting of these conditions in electronic health records (EHR) [7,8]. RFS incidence is currently uncertain, since the lack of a universally accepted definition of RFS hampers the interpretation of epidemiological studies [9]. Multi-center and prospective studies in the inpatient medicine area indicate an incidence of approximately 8–14% in malnourished inpatients [9,10,11]. RFS is not specifically documented in EHRs, since there is no specific International Statistical Classification of Diseases and Related Health Problems (ICD) ICD-10 Label for this condition. It is mostly ICD coded as E87.8 (“other electrolyte disturbances”) or E83.3 (“disturbances of phosphate metabolism”), with E87.8 being the more specific label recommended for RFS coding. RFS imposes relevant risk to patients, because its occurrence during refeeding increases morbidity and mortality and adequate therapy considerations need to be applied [10,12]. Clinical decision support systems (CDSS) can improve medical practitioner performance and care [13,14]. To improve RFS recognition and management, we retrospectively developed and prospectively investigated a clinical decision support system (CDSS) in a before/after cohort study. The CDSS supports physicians in diagnosing and managing RFS by automatically evaluating laboratory data and encoded items in the EHRs in University of Leipzig Medical Centre (ULMC), Germany. The CDSS gives therapy recommendations for suspected RFS patients. The scope of this publication is the (i) retrospective diagnostic algorithm development and features of the RFS CDSS, (ii) prospective feasibility testing in real-life inpatient care, (iii) integration into clinical workflow and (iv) limitations of the CDSS.

## 2. Materials and Methods

### 2.1. Study Design

**Figure 1:**
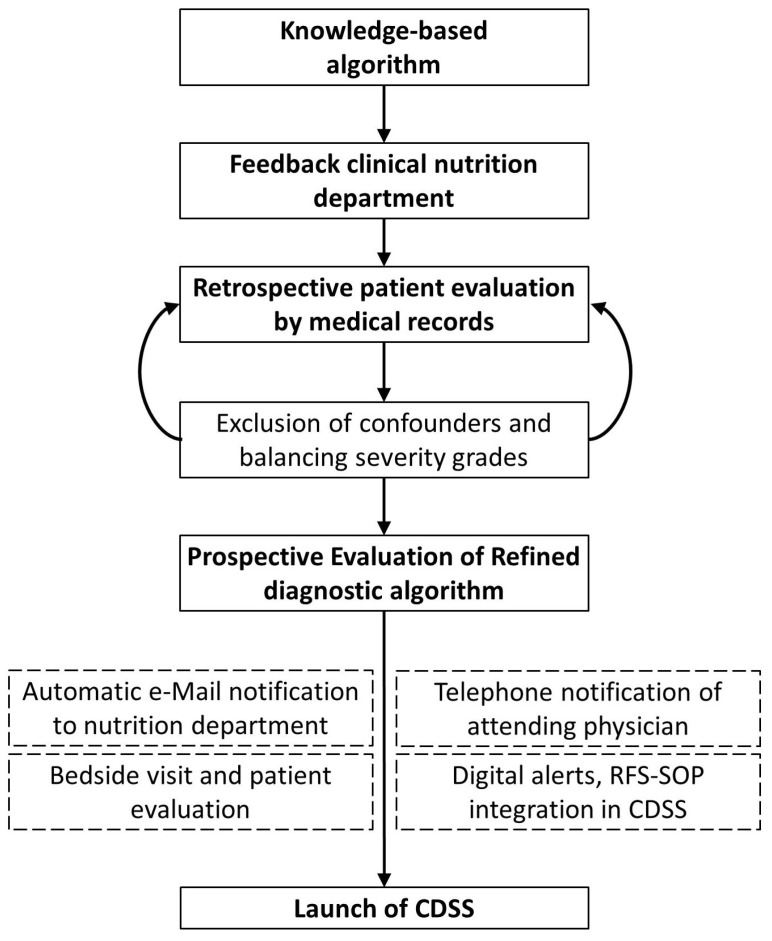
Study design for retrospective interdisciplinary refeeding syndrome (RFS) recognition algorithm development through an iterative revision process and integration into clinical workflow, followed by six months of prospective feasibility testing (clinical decision support system = CDSS, OPS = operational procedure codes, refeeding syndrome = RFS, SOP = standard operating procedure).

We designed a knowledge-based diagnostic algorithm for refeeding syndrome (RFS) using a retrospective dataset for testing purposes and discussed the algorithm with the clinical nutrition department. We integrated the derived algorithm for clinical users into a web-application-based clinical decision support system (CDSS) with the clinical information system that displays traffic light flagging symbols as non-disruptive alerts in the respective patients electronic health record (EHR), as well as RFS severity with follow-up and referral recommendations in a pop up window. The CDSS was integrated into clinical workflow by also notifying the clinical nutrition department about the susceptible RFS patient by automatically generated e-mails. The department carried out bedside consultations to patients after notification. The CDSS was prospectively investigated in a six-month feasibility study in real-life inpatient care. During feasibility testing, the physician in charge was additionally informed about detected patients by telephone by the study physician (Figure 1). Part of the prospectively assessed data was already published in a review paper about the diagnostic potential that CDSS applications yield in RFS recognition improvement [15]. The study was approved by the Ethics Committee of the Medical Faculty of the University of Leipzig (No. 214/18-ek).

### 2.2. Development and Refinement of Diagnostic Algorithm

The algorithm was derived by an iterative revision process. Initially, the study physician designed a knowledge-based framework following the American Society of Parenteral and Enteral Nutrition (ASPEN) consensus diagnostic criteria for RFS (Figure 1). The AS-PEN criteria describe RFS as a decrease in any or several of the serum electrolyte concentrations of phosphate, potassium and/or magnesium by 10–20% (mild), 20–30% (moderate) or >30% (severe) and/or organ dysfunction within five days of newly introduced food intake in a malnourished patient [3]. The framework applies IF-THEN rules to phosphate, potassium and magnesium concentrations. In case of electrolyte concentration reduction of 10–20% (mild RFS) the algorithm also considers digitally available information (items) about reduced food intake to increase detection specificity. These items are collected upon admission and documented as checkbox information into the EHR. Potential challenges and disadvantages were identified and discussed with the clinical nutrition department. The derived algorithm was tested on a retrospective dataset (for dataset description, see Section 2.4). RFS diagnosis correctness and severity were evaluated in the retrospective patient cohort by manual evaluation of the patients’ medical records and laboratory data (Figure 1). 

Patient identification solely based on this raw framework identifies a proportion of patients with other underlying conditions with similar alterations in electrolyte concentrations (e.g., hypophosphatemia induced by renal replacement therapy for renal failure). We incorporated exclusion criteria as a refinement strategy for the algorithm that address these underlying conditions by using patient data, encoded operational procedure codes (OPS) and laboratory data. Patients under the age of 18 were excluded by their age derived from their birth date. Operational procedure codes are a German coding system to systematically record all operations and treatment procedures applied during a patient’s hospitalization for billing purposes. OPS are usually encoded in the EHR within 24 h after the treatment procedure was performed. The CDSS screened for the presence of OPS that indicated the performance of procedures that cause RFS-like electrolyte disturbances (e.g., renal replacement therapy; for detailed information on exclusion criteria, see Table S2 in the Supplement). If these OPS codes occurred in the EHR before the CDSS registered the RFS-susceptible electrolyte imbalance, the otherwise issued alert was suppressed. This was intended to avoid alert fatigue.

In a similar fashion, the CDSS suppressed alerts for hypophosphatemia of other origin then RFS. Alert suppression was performed in the case of detection of ketones in urine (diabetic ketoacidosis), paracetamol in serum (paracetamol intoxication), phosphate or magnesium concentrations above the upper reference range (severe acute or chronic renal failure), parathyroid hormone concentrations above the upper reference range (hyperparathyroidism) or lung or neuroendocrine malignancies (secretion of parathyroid hormone-related peptide) in the patients’ laboratory data before RFS-susceptible electrolyte imbalance (for detailed information on used cut-offs and lab values for exclusion, see Table S2 in Supplement). These comorbidities are known to induce hypophosphatemia without association to RFS [16,17,18,19,20].

**Figure 2:**
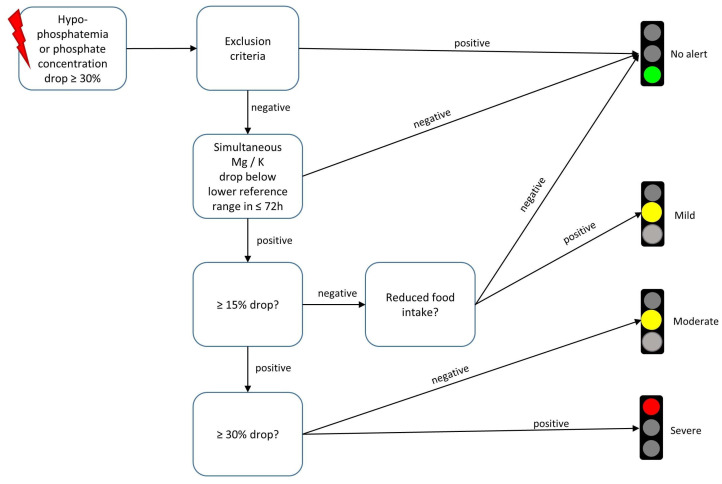
Three-step-wise evaluation of electrolyte concentrations and exclusion criteria for refeeding syndrome detection of a knowledge-based refeeding syndrome recognition algorithm. The triggering event (symbolized by red lightning pictogram) that initiates algorithmic electrolyte concentration evaluation is the detection of Hypophosphatemia or a phosphate concentration decrease of at least 30%.The algorithm is embedded in a clinical decision support system that sends digital alerts to affected patients’ electronic health records. The digital alerts are displayed as traffic lights. The color coding follows the respective severity of the electrolyte disturbance. Clicking on the traffic light symbol opens up therapy and referral recommendations tailored to refeeding syndrome severity (Mg = magnesium; K = potassium, h = hours).

The final diagnostic algorithm performs patient identification in a three-step process: (1) initiation through patients with hypophosphatemia (<0.84 mmol/L) or decrease in phosphate concentration ≥30%, (2) automatic exclusion of patients with hypophosphatemia of other origins by matching with OPS codes and laboratory values in the EHR and (3) electrolyte concentration evaluation for RFS severity (for a schematic representation, see Figure 2).

### 2.3. CDSS Evaluation and Clinical Workflow

The CDSS was implemented into an SAP-based clinical information system. Technical requirements for the CDSS have already been described [21]. Non-disruptive alerts are generated by the CDSS and represented as traffic light symbols for concerning patients. Clicking on the symbol opens a pop-up window, which further displays RFS severity, current phosphate concentration and RFS severity associated clinical information (for presented clinical information see Table S1 in Supplementary Material). Severity grading follows traffic light color coding schemes, with green indicating no RFS, yellow indicating mild to moderate RFS and red indicating severe RFS. Alert suppression in case of exclusion criteria being met is performed by the CDSS presenting a green traffic light. The CDSS also automatically notifies the clinical nutrition department about details on the potential RFS patient via e-mail alert. During the six-month feasibility study, the CDSS additionally notified the study physician, who notified the attending physician at the ward about the possible RFS patient by telephone and conducted a short questionnaire (see questions below).

(1) Are you familiar with RFS?
(2) Which laboratory value would you use to diagnose RFS?
(3) Did the patient show a new symptom today?
(4) Did you already consider a referral to the clinical nutrition department and if not, why?

Following the CDSS alert, a physician or a clinical nutritionist carries out a bedside consultation to confirm RFS diagnosis. During the consultation the nutritionist records the nutrition-related medical history looking for RFS risk factors, evaluates malnutrition and gives written recommendations for further patient-tailored monitoring and nutrition management to the attending physician. RFS risk factors are evaluated by applying criteria of the British National Institute for Health and Care Excellence (NICE) criteria [22], which are commonly used for identifying patients at risk for RFS in clinical practice. Malnutrition severity is evaluated by applying the Nutritional Risk Screening 2002 (NRS). An NRS score ≥ 3 indicates a necessity for nutritional intervention [23]. 

### 2.4. Data Analysis

**Figure 3:**
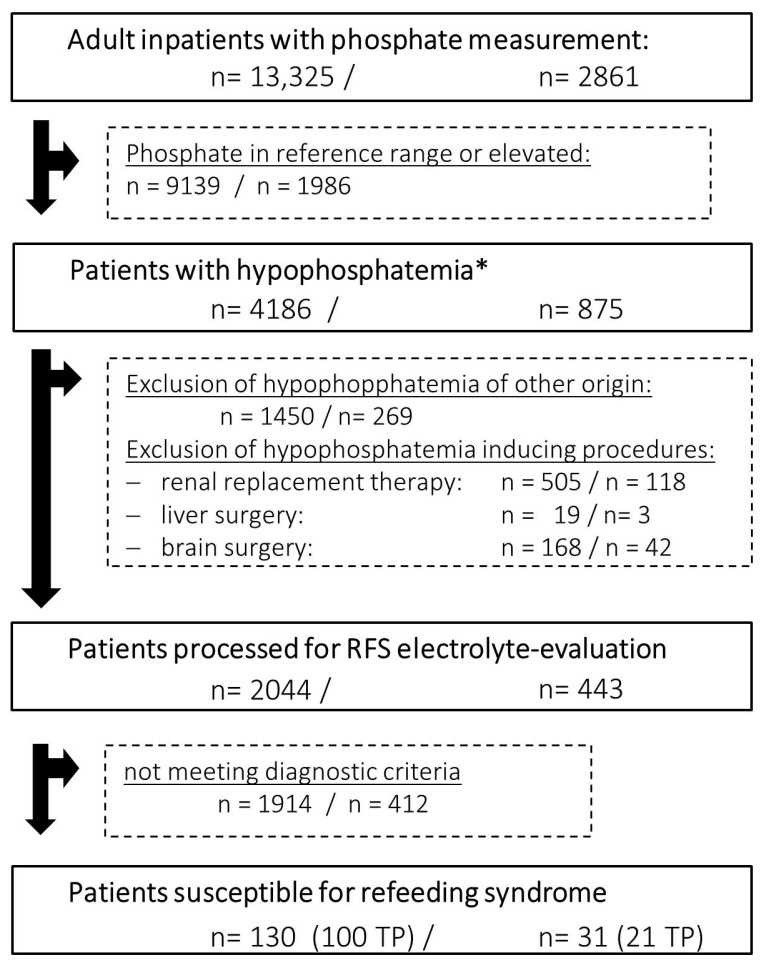
Retrospective (**left**) and prospective (**right**) data analysis of all inpatients with phosphate measurements processed by clinical decision support system (CDSS) for refeeding syndrome (RFS) detection. Exclusion criteria are applied before the CDSS performs electrolyte concentration evaluation for RFS diagnosis considering phosphate, potassium and magnesium concentrations (* <0.84 mmol/L, RFS = refeeding syndrome, TP = true positive).

The RFS algorithm was developed using retrospective data from adult inpatients with phosphate measurements from January 2019 to June 2021 (*n* = 13,325 cases, Figure 3, left) at the ULMC. The dataset included:

- Laboratory data: concentrations of phosphate, potassium, magnesium, paracetamol, parathyroid hormone, parathyroid hormone-related peptide and total ketone bodies. All parameters were measured in serum or full blood samples besides for ketones, which were also measured in urine samples
- Encoded items in the EHR: age, sex, reduced food intake at admission, body mass index (BMI), ward category (intensive care units vs. normal wards), OPS codes regarding dialysis, brain surgery, liver surgery, nutritional referrals and ICD-10-codes for RFS (E83.3 and E87.7)
- Primary outcome in prospective evaluation: can the CDSS facilitated RFS diagnosis be confirmed by clinical experts?

The CDSS processed the same data points of adult inpatients during prospective testing from December 2021 to May 2022 (*n* = 2861, Figure 3, right). RFS diagnosis correctness and severity were evaluated in the retrospective patient cohort by manual evaluation of the patients EHR and laboratory data. The clinical nutrition department confirmed RFS diagnosis during the prospective feasibility study during bedside consultations. The proportion of correctly identified RFS patients was the primary outcome for the feasibility testing. We performed data handling in R 4.1.3 [24]; for plotting, we used the R library ggplot2 [25].

## 3. Results

The retrospective testing of the refined diagnostic algorithm for refeeding syndrome (RFS) identified 130 patients in 30 months, of whom 100 patients suffered from RFS (true positive patients, TP), whereas 30 patients showed electrolyte imbalances similar to RFS but with other etiology (false positive patients, FP), resulting in 77% correctly identified patients with RFS. In the prospective investigation, the clinical decision support system (CDSS) detected 31 patients suspected of having RFS. The clinical nutrition department verified RFS diagnosis in 21 patients, resulting in 67% correctly identified patients with RFS. In 13 of these 21 patients the medical team caring for these patients did not recognize RFS yet. FP patients were mostly detected due to renal replacement therapy or brain surgery with pending OPS coding, intracerebral hemorrhages or head injuries or end-stage liver disease. These procedures and comorbidities have already been described as causing electrolyte imbalances resembling RFS without reference to nutrition status [16,17,18,19,26]. These confounders are included in the clinical information presented by the CDSS user interface (see Table S1 in the Supplementary Materials). Prospective TP patients presented with median Nutritional-Risk-Screening (NRS) Score of 4, indicating need for nutritional intervention, while prospective FP patients presented with a NRS of 2, indicating a need for a follow-up reevaluation of malnutrition (Table 1). Median BMI for men and women ranged from 20–25 kg/m^2^, depending on the specific cohort investigated, indicating average, normal weights. National Institute for Health and Care Excellence (NICE) criteria were positive for 52% and 71.4% in the true positive patients of the retrospective and prospective cohorts. NICE criteria were positive for one patient in both false positive cohorts, respectively (Table 1). Coding for phosphate-related electrolyte disturbances was low overall, with 23 patients (17%) in the retrospective cohort and 8 patients (23%) in the prospective cohort. Most TP patients suffering from RFS were cared for at normal wards (60% and 76% in the retrospective and prospective cohort, respectively). 

Telephone notification of physicians attending to susceptible RFS patients resulted in 24 conducted short questionnaires. In the missing seven cases, the attending physicians were not available for telephone notification. From 24 interviewed physicians, 17 attended to confirmed, true positive RFS patients. The telephone notification of the attending physician during the feasibility study revealed that a large proportion of physicians were not familiar with RFS or laboratory values used for diagnosis (11/24, Table 2, line 2). Three of the physicians stated they already included the clinical nutrition department into the management of the patient during the interview. The reasons for not including the clinical nutrition department in the case before CDSS intervention were highly variable and did not fit into broader categories. Some repeated reasons are presented in the legend of Table 2.

## 4. Discussion

This study describes the development of a clinical decision support system (CDSS) for refeeding syndrome (RFS) recognition and its integration into clinical workflow, providing a proof of concept for CDSS-facilitated diagnosis of RFS. The CDSS was able to recognize patients suffering from RFS with an acceptable false positive rate (retrospective 23% and prospective 33%). The utilization of the CDSS lead to an RFS diagnosis in 13 out of 21 (62%) true positive patients in the prospective cohort. It also resulted in several improvements in patient-related care and documentation aspects, e.g., in a rise of RFS-specific medical coding (retrospective cohort E87.7 once coded in 30 month vs. prospective cohort E87.8 four times coded in six month) and in a doubled rate of nutritional therapy referrals in true positive patients (retrospective TP 33% vs. prospective TP 71%). To the best of our knowledge, this is the first described and clinically utilized CDSS for RFS recognition.

A large proportion of physicians is not familiar with RFS, making it a neglected condition. In a current questionnaire among 281 clinical physicians in Germany with a case vignette about a malnourished patient developing RFS, only 14% of the physicians correctly recognized RFS [27]. This unfamiliarity was also recognizable in international audits, which evaluated current practice in prescription and management of parenteral nutrition in the United Kingdom and New Zealand. More than half of the audit participants were not able to identify patients at risk for RFS, despite electrolyte monitoring according to clinical nutrition guidelines [28,29]. Our short, telephone-based questionnaire demonstrated this unfamiliarity as well, with 8 out of 24 interviewed physicians being unfamiliar with RFS, and 11 out of 24 being unfamiliar with lab measurements used for its diagnosis (Table 2). In consequence, the clinical nutrition department drew up a standard operating procedure (SOP) for RFS treatment to answer the educational demand under clinical users. The SOP summarizes information on medications currently used at University of Leipzig Medical Centre (ULMC), their appropriate dosage and who to contact at the clinical nutrition department in case of further questions. The RFS SOP was integrated into the CDSS user interface as part of the clinical information by hyperlink. The SOP was accessed 42 times in eight weeks following the launch of the CDSS, mirroring the success of the CDSS-feasibility testing.

RFS causes non-specific symptoms that many other frequent comorbidities without any root causes in nutritional status also show (e.g., cardiac arrhythmias and edemas are frequent symptoms of heart insufficiency). Most CDSSs detected that RFS-patients showed no new symptoms on the day of diagnosis (16/24, Table 2, line 3). RFS diagnosis solely based on clinical presentation is, therefore, highly challenging [7]. A universal definition for RFS is still not available and this hampers not only the comparability of scientific evidence and epidemiologic reporting but also the recognition of the condition [9,30]. Current recommendations for RFS management propose an initial risk assessment of patients and measurement of electrolyte concentration regarding phosphate, potassium and magnesium before nutrition initiation [3,31,32]. Initial risk assessment before nutrition initiation requires physician awareness about the patients’ nutrition status. Malnutrition prevalence in inpatients is high, with reporting rates between 25–40%, which places them at risk of RFS during nutrition initiation [33,34,35]. However, in contrast to the considerably high reporting rates for malnutrition prevalence, malnutrition diagnosis is reported in only about 4–5% of hospitalized cases, as Tobert et al. revealed in their analysis of 105 US academic medical centers in 2018 [36]. Vest et al. demonstrated in a retrospective cohort study that physicians show a much more frequent recognition of malnutrition in patients with a BMI below 18.5 kg/m^2^. Malnutrition was diagnosed in 43% of these patients, while patients with a BMI higher than 18.5 kg/m^2^ only received a malnutrition diagnosis in 26% [8]. The median BMI within our retrospective and prospective cohort were above 18.5 kg/m^2^ (Table 1), which we understand to be contributing to the underestimation of RFS risk in these patients. 

CDSS interventions in healthcare have shown positive effects on patient-related outcomes [37], morbidity prevention [38], prescribing medication behavior [39], practitioner performance [40] and improvements in care-related processes [13]. Yet, a recent meta-analysis by Ronen et al. did not find conclusive evidence that CDSSs change the behavior of health practitioners to adopting desired practices in the inpatient setting [41]. Another meta-analysis by Moghadam et al. investigated the effects of a CDSS for medication prescription on patient outcomes and practitioner performance. They described positive effects conveyed by CDSS depending on disease type or clinical field they were utilized in and concluded this to be one of the reasons for conflicting results about CDSS’s effects [39]. The utilization of CDSS in clinical nutrition is not yet common. 

However, a few studies have shown promising results in clinical nutrition settings, such as improved calorie and protein intake in critically ill hematology patients [42] or improved glycemic control in neurotrauma intensive care patients [43]. We interpret these results as mounting evidence that clinical nutrition is a field in which the utilization of CDSS has potential to support health care providers and facilitate improvements in care. Due to the widespread occurrence of malnutrition and its associated risk for RFS in most medical specialties, CDSSs for RFS recognition have a promising diagnostic yield and have broad applicability. In our cohort, 13 out of 21 medical teams caring for patients suffering from RFS did not recognize the condition before CDSS intervention. ICD 10 coding for RFS remained low overall but showed an increase for RFS recommended code E87.8 (retrospective 1% [n = 1] vs. prospective 19% [n = 4]). Three physicians stated during the telephone questionnaire that they were familiar with RFS but not having considered it for the patient just yet. We interpret this as first evidence that the investigated CDSS supported health practitioners to evaluate the patient for a diagnosis they had not previously considered by using already-collected clinical data. 

A barrier for higher CDSS impact is a commonly reported low uptake by health care providers [44]. We combined our CDSS intervention with an automated involvement of the clinical nutrition department to confirm diagnosis during feasibility testing. This combination turned out to be an attractive feature of the integration of the CDSS into clinical workflow. The responsibility to ensure clinical action to patients at risk is shared between the attending physician at the ward as well as the nutrition department. Another explorative study by Haase-Fielitz et al. in 2020 that investigated the effect of a combination of an electronic alert system for the detection of acute kidney injury with a nephrology consultation at the same day in comparison to standard care revealed similar effects. The intervention arm regained baseline renal function and documented acute kidney injury diagnosis significantly more often, while kidney-related complications were reduced [45]. The positive effects of this combined approach on clinical outcomes needs to be verified in larger controlled randomized trials, but is deemed to be an attractive feature for CDSS implementation into clinical practice.

## 5. Limitations

Due to a small number of cases in the study period, randomization could not be performed. In addition to that, diagnostic performance of the clinical decision support system (CDSS) underlying refeeding syndrome (RFS) recognition algorithm can currently not be determined. Classical performance measurements, such as the positive predictive value, sensitivity or specificity, all rely on complete information on true and false negative patients. Complete RFS-related electrolyte measurements in all malnourished inpatients during their hospitalization after nutrition initiation would be needed to estimate these measures. This information was not available to us. Electrolyte monitoring in clinical practice mainly focuses on potassium and sodium. The to us accessible retrospective dataset consisted of 73,616 EHRs. It contained 13,325 adult patients with blood withdrawals and phosphate measurements, which are only about 18% of cases. We were, therefore, not able to estimate all the unnoticed cases due to incomplete electrolyte measurements, RFS unspecific and underperformed coding or subclinical symptom presentation of RFS-related complications. The RFS detection algorithm performance needs to be tested by a large randomized controlled trial.

## 6. Conclusions

This publication describes the development and feasibility testing of a laboratory and electronic health record data-based CDSS for RFS detection, combined with a follow-up visit by the nutrition department to ensure CDSS uptake. Without CDSS diagnosis support, RFS remains an often-overlooked condition by physicians. This study provides a proof of concept for CDSS-facilitated diagnosis of RFS and improvement in care in the inpatient setting. Its effect on clinical outcomes needs further investigation in large randomized-controlled trials.

## Figures and Tables

**Table 1:** Patient characteristics of the retrospectively and prospectively investigated cohorts, stratified for true positive and false positive patients. Data reported as relative and absolute frequencies or median (MD) with the first and third quartile.

	Retrospective Cohort		Prospective Cohort	
	True Positive (n = 100)	False Positive (n = 30)	True Positive (n = 21)	False Positive (n = 10)
Sex [m/f]	58%/42%	46%/53%	38%/62%	80%/20%
Age [years]	65 [58; 76]	66 [50; 77]	65 [56; 74]	67 [59; 74]
BMI [kg/m^2^]				
male	24.1 [20.8; 26.9]	22.9 [22; 24.6]	21.6 [17.4; 21.6]	23.6 [21.7; 25.9]
female	20.3 [18.3; 23.9]	25 [20.9; 29.8]	20 [19.1; 24.5]	23.7 [23.3; 24.1]
Red. food intake				
Yes	56% (56)	40% (12)	47% (10)	60% (6)
No	40% (40)	53% (16)	53% (11)	40% (4)
NA	4% (4)	8% (2)	-	-
NRS-2002	4 [4; 5] (n = 28)	4 [1; 5] (n = 4)	4 [3; 5] (n = 20)	2 [2; 2] (n = 7)
Positive NICE criteria	52% (52)	3.3% (n = 1)	71.4% (n = 15)	10% (n = 1)
Alert-severity				
mild	46% (46)	10% (3)	9.5% (2)	60% (6)
moderate	30% (30)	53% (16)	57.5% (12)	30% (3)
severe	24% (24)	37% (11)	33% (7)	10% (1)
Phosphate [mmol/L]	0.53 [0.46; 0.58]	0.55 [0.47; 0.61]	0.48 [0.43; 0.53]	0.66 [0.55; 0.75]
Potassium [mmol/L]	3.04 [2.78; 3.31]	2.9 [2.8; 3.3]	3 [2.78; 3.09]	3.09 [3.04; 3.32]
Magnesium [mmol/L]	0.71 [0.6; 0.78]	0.76 [0.63; 0.82]	0.71 [0.63; 0.76]	0.66 [0.6; 0.74]
**ICD-10**				
E83.3	21% (21)	26% (8)	19% (4)	0
E87.7	1% (1)	0	19% (4)	0
No coding	78% (78)	74% (22)	81% (17)	100% (10)
Ward				
ICU	40% (40)	50% (15)	24% (5)	10% (1)
Non-ICU	60% (60)	50% (15)	76% (16)	90% (9)
Nutrition referral				
yes	33% (33)	10% (3)	71% (15)	60% (6)
no	67% (67)	90% (27)	29% (6)	40% (4)

BMI = body mass index, f = female, ICU = intensive care unit, ICD-10 = International Statistical Classification of Diseases and Related Health Problems, NA = not available, National Institute for Health and Care Excellence = NICE, NRS-2002 = Nutritional-Risk-Screening 2002, m = male, Red. Food Intake = reduced food intake.

**Table 2:** Telephone short questionnaire performed at the time of alert. Results from 24 attending physicians (n = 24) responsible for patients with refeeding syndrome diagnosis proposed by the clinical decision support system at their ward, including seven false positive patients.

Question	Given Answers	Count
Are you familiar with refeeding syndrome?	yes	16
	no	8
Which laboratory value would you use to diagnose refeeding syndrome?	Unknown	11
	Glucose	1
	Phosphate	8
	Electrolytes	4
Does the patient show a new symptom today?	Diarrhea	1
	Abdominal pain	2
	Weakness	2
	Weight loss	1
	Hypokalemia	1
	No new symptom	16
	Not available	1
Did you already consider a referral the clinical nutrition department?	Yes	3
	Not available	1
	No *	20

* repeated reasons for omitted nutrition department involvement: attending physician is familiar with RFS, but did not think about it until telephone notification (3×), patient-related incompliance (2×), new patient that has not been seen yet (2×).

## Data Availability

Our data are available upon request. Although data are pseudonymized, details such as the combination of sex, age, ward category, laboratory data and period of assessment at University of Leipzig Medical Centre (ULMC) could potentially be used to identify individual cases and patients. Therefore, in accordance with the General Data Protection Regulation, we are restrained regarding the public release of our dataset. When administrative and legal requirements are met, trusted research institutions may request data access through the current director of the Institute of Laboratory Medicine, Clinical Chemistry and Molecular Diagnostics Leipzig (MB-sek@ilm@medizin.uni-leipzig.de) or the AMPEL project directly (MB-ilm-ampel@medizin.uni-leipzig.de).

## Supplementary Materials

The following supporting information can be downloaded at: https://www.mdpi.com/article/10.3390/nu15173712/s1; Table S1: Presented clinical information in regard to detected refeeding syndrome severity. Standard operating procedure for refeeding syndrome management following clinical guidelines was integrated by hyperlink into the user interface of the clinical decision support system; Table S2: Exclusion criteria. Overview of used operational procedure codes (OPS) billing codes and laboratory data with cut offs that make other origin, such as electrolyte imbalances, more likely than refeeding syndrome.
