# Supplementary material for: Development, Design and Utilization of a CDSS for Refeeding Syndrome in Real Life Inpatient Care—A Feasibility Study
Source: Nutrients. 2023 Aug 24;15(17):3712. doi: 10.3390/nu15173712 (PMC10490138; doi:10.3390/nu15173712)
Supplement: Supplementary file 1 [file nutrients-15-03712-s001.zip › nutrients-2569846-supplementary.pdf]

Nutrients 2569846 - Heuft, RFS – Supplementary material

Table S1. Presented clinical information in regard to detected refeeding syndrome severity. Standard operating procedure for refeeding syndrome management following clinical guidelines was integrated by hyperlink into the user interface of the clinical decision support system.

RFS severity	Clinical information
Mild	Reduced food intake in combination with relevant hypophosphatemia was detected. Laboratory chemistry suggests refeeding syndrome. Has nutrition been introduced after several days of food abstinence? If yes, it is recommended to determine magnesium and potassium to complete the diagnosis and a patient referral to the nutrition team. Electrolyte imbalances resembling refeeding syndrome have also been described in traumatic brain injury and after liver surgery. Please check plausibility.<br>(Hyperlink to Standard Operating Procedure for RFS)
Moderate	Laboratory diagnosis of refeeding syndrome. Daily checks of potassium, magnesium, and phosphate until electrolyte balance is achieved are recommended and a patient referral to the nutrition team. Electrolyte imbalances resembling refeeding syndrome have also been described in traumatic brain injury and after liver surgery. Please check plausibility.<br>(Hyperlink to Standard Operating Procedure for RFS)
Severe	Laboratory diagnosis of severe refeeding syndrome, fatal cases have been described in the literature. Daily checks of potassium, magnesium, and phosphate until electrolyte balance is achieved are recommended and that consultation with a nutritionist be sought immediately. Electrolyte imbalances resembling refeeding syndrome have also been described in traumatic brain injury and after liver surgery. Please check plausibility.<br>(Hyperlink to Standard Operating Procedure for RFS)

Table S2. Exclusion criteria. Overview of used operational procedure codes (OPS) billing codes and laboratory data with cut offs that make other origin of like electrolyte imbalances more likely than refeeding syndrome.

**Exlcusion Criteria**	**Codes or Cut-offs**
Age	<18 years
Procedures by OPS	
Renal replacement therapy	8-831.5; 8-854; 8-854.2; 8-854.3; 8-854.4; 8-854.5; 8-854.6; 8-854.60 - 8-854.69; 8-854.6a; 8-854.6b; 8-854.6c; 8-854.7; 8-854.70 - 8-854.79; 8-854.7a; 8-854.7b; 8-854.7c; 8-854.8; 8-854.x; 8-854.y?
Brain surgery and interventions	5-010 up to and including 5-018<br>5-02 up to and including 5-02.9
Liver surgery	5-500 up to and including 5-509
Laboratory data	
Serum samples	Paracetamol > 0 µg/ml; Parathyroid hormone > 6,9 pmol/l or <1,6 pmol/l; Total ketone bodies > 120 umol/l; 3-hydroxybutyrate > 74 umol/l; Phosphate > 1,45 mmol/l; Magnesium > 0,99 mmol/l
EDTA samples	Parathyroid hormone related peptide > 1,5 pmol/l; Parathyroid hormone > 6,9 pmol/l or <1,6 pmol/l
Urine samples	Total ketone bodies > 0 mmol/l
